# Aetiology of paediatric pneumonia with effusion in the Dominican Republic and the potential impact of pneumococcal conjugate vaccines

**DOI:** 10.15172/pneu.2014.4/413

**Published:** 2014-12-01

**Authors:** Jesús Feris-Iglesias, Josefina Fernández, Jacqueline Sánchez, Fabiana Pimenta, Chabela Peña, Hilma Coradin, Eddy Perez-Then, Maria Peinado, Angélica Floren, Teresa Del Moral, Dean Erdman, Maria da Gloria Carvalho, Jennifer R. Verani

**Affiliations:** 12 Department of infectious Diseases, Dr. Robert Reid Cabral Children’s Hospital, Santo Domingo, Dominican Republic Ave. Abraham Lincoln 2, ZP 0002; 22 Centers for Disease Control and Prevention, Atlanta, GA USA; 32 University of Miami, Miami, FL USA

**Keywords:** pneumonia, pleural effusion, *Streptococcus pneumoniae*, pneumococcal vaccines, Dominican Republic

## Abstract

Pleural effusion is a serious complication of pneumonia, and *Streptococcus pneumoniae* is a leading cause. We describe the aetiology of pneumonia with effusion among children in the Dominican Republic before the introduction of the 13-valent pneumococcal conjugate vaccine (PCV) in 2013 and the performance characteristics of a rapid immunochromatographic test (ICT) for detecting *S. pneumoniae* in pleural fluid. From July 2009 to June 2011, we enrolled children <15 years old admitted with pneumonia and pleural effusion to Robert Reid Cabral Children’s Hospital, Dominican Republic. Pleural fluid was tested by culture, polymerase chain reaction (PCR) for bacterial (*S. pyogenes, S. pneumoniae*) and viral (respiratory syncytial virus and human rhinovirus) pathogens, and by ICT for *S. pneumoniae*. We calculated the performance of ICT and culture compared with PCR. Among 121 cases, the median age was 31 months (range 1 week to 14 years). Pleural fluid culture (*n* = 121) and PCR testing (*n* = 112) identified an aetiology in 85 (70.2%) cases, including 62 *S. pneumoniae* (51.2%) and 19 *Staphylococcus aureus* (15.7%). The viruses tested were not detected. The most prevalent pneumococcal serotypes were 14 (*n* = 20), 1 (*n* = 13), and 3 (*n* = 12). Serotype coverage of the 10- and 13-valent PCVs would be 70.5% and 95.1%, respectively. The sensitivity of point-of-care ICT was 100% (95% confidence interval [CI] 94.1%–100%), while specificity was 86.3% (95% CI 73.7%–94.3%). *S. pneumoniae* caused more than half of paediatric pneumonia with effusion cases; introduction of PCV in the Dominican Republic could reduce the burden by 36–49%. ICT is a practical, valid diagnostic tool for clinical care and surveillance in settings with limited laboratory capacity.

## 1. Introduction

Pneumonia is the leading cause of death among children worldwide [1]. Pleural effusion is a serious complication of pneumonia affecting up to 40% of children hospitalised with pneumonia [2]. Parapneumonic effusion, also known as pneumonia with effusion, is associated with longer hospitals stays, more intensive care, and higher rates of morbidity [3, 4]. In the Dominican Republic, pleural effusions are frequent and complicate 38% of admissions for pneumonia at Robert Reid Cabral Children’s Hospital (RRCCH), which is a large referral hospital in the capital city of Santo Domingo; one of every two admissions to the infectious disease service is due to pneumonia with effusion [5].

While various pathogens can lead to pneumonia with effusion in children, the most common causes are bacterial — particularly *Streptococcus pneumoniae* [6]. Data on the aetiology of parapneumonic infections can help guide clinical management as well as policies for preventive interventions such as the pneumococcal conjugate vaccine (PCV). Yet establishing the cause of pneumonia with effusion can be challenging. Culture is insensitive, and prior antibiotic use further decreases its yield [7]. While more sensitive diagnostic tests such as polymerase chain reaction (PCR) and antigen detection tests exist, they are not widely available in resource-constrained settings. Consequently there are relatively limited data from low- and middle-income countries on the principle causes of pneumonia with effusion.

The 13-valent PCV was introduced in the routine infant immunisation program in the Dominican Republic in August, 2013. Prior to the introduction, we undertook a study of the epidemiology of pneumonia with effusion among children in the Dominican Republic to inform PCV policy decisions. We used culture, PCR and a rapid Immunochromatographic test (ICT) (BinaxNOW®; Alere Inc, USA) to determine predominant aetiologies and estimate the proportion of disease that would be prevented through PCV introduction in the routine infant immunisation program.

## 2. Methods

### 2.1 Patients and sample collection

Surveillance for pneumonia with effusion was conducted at RRCCH from July 2009 to June 2011. All children less than 15 years old admitted with history of fever and/or measured temperature ≥38 °C on admission, tachypnoea (defined as a respiratory rate >30 per minute in children less than 8 years old and >25 in children aged 8 to 16 years), and radiological evidence of pleural effusion large enough to require thoracocentesis as a diagnostic or therapeutic procedure were eligible to participate. After obtaining informed written consent from a parent or guardian (and written assent from children ≥12 years old), clinical and epidemiologic data were gathered from medical records and parental interview using standardised forms, and thoracocentesis was performed. Pleural fluid samples were sent immediately to the microbiology laboratory of RRCCH for Gram stain, culture, antimicrobial susceptibility testing, and point of care ICT for *S. pneumoniae*. Remaining pleural fluid was frozen at −70 °C and sent to the United States (US) Centers for Disease Control and Prevention (CDC) for molecular testing and repeat of the ICT.

### 2.2 Laboratory methods

All pleural fluids were cultured on blood, chocolate and MacConkey agar plates for bacterial isolation, followed by identification according to recommendations in the Manual of Clinical Microbiology [8]. *S. pneumoniae* isolates were identified by alpha-haemolysis, optochin susceptibility and bile solubility. Antimicrobial susceptibility tests were conducted using disc diffusion and E-test for penicillin and cefotaxime [9]. The ICT test *S. pneumoniae* BinaxNOW® was performed according to manufacturer’s instructions as a point of care test at the hospital laboratory. When available, remaining specimen was frozen at −70 °C and sent to CDC for ICT re-testing and performance of PCR for bacterial (*S. pyogenes* and *S. pneumoniae*) and viral (respiratory syncytial virus [RSV] and human rhinovirus) pathogens.

Just before bacterial DNA extraction, 200 µl volumes of the pleural fluid samples were manually transferred into 1.5 ml cryotubes containing 100 µl of Tris-EDTA buffer with 0.04 g/ml lysozyme and 75 U/ml mutanolysin (Sigma Chemical Co., USA). The mixture was incubated for 1 h at 37 °C followed by addition of 20 µl of proteinase K. After mixing briefly with a vortex machine, 400 µl of lysis buffer (Qiagen, USA) was added. After completion of the lysis step, the extraction process followed the manufacturer’s procedures using the NucliSENS® easyMAG® automated nucleic acid extraction system (Biomerieux, USA). Bacterial DNA extracts were eluted in 100 µl of elution buffer. Viral total nucleic acids were recovered from separate 200 µl volumes of pleural fluid placed directly in NucliSens® Lysis Buffer (Biomerieux) and extracted as described above. *S. pneumoniae* and *S. pyogenes* DNA was detected using real-time PCR assays targeting the *lytA* and *spy* genes, respectively [10, 11]. For *lytA*-positive specimens, pneumococcal serotype deduction was achieved using a sequential multiplex PCR strategy for clinical samples [12, 13]. RSV [14] and human rhinovirus [15] were tested by real-time reverse transcription PCR assays as previously described. Positive controls were run concurrently for each specific PCR assay as well as a control reaction with RNAseP human gene performed independently with each sample to check for the presence of inhibitors [10].

### 2.3 Data analysis

Data were analysed using SAS (v 9.3, SAS Institute Inc., Cary NC). We described the frequencies of clinical characteristics and aetiologies, and assessed the proportion of disease preventable by available 7-, 10- and 13-valent PCV formulations. Aetiology was determined based on positive results by culture and/or PCR. To evaluate the performance of the ICT to detect *S. pneumoniae* in pleural fluid, we calculated sensitivity, specificity, positive and negative predictive values with 95% confidence intervals (CI) using PCR results as the gold standard.

### 2.4 Ethical approval

The study was conducted in accordance with the Helsinki Declaration. The protocol was reviewed and approved by the Ethics Committee and Institutional Review Board of Fundacion Dominicana de Infectologia, Inc. (Approval reference no: 2008-05). The Human Subject Research Protection Office of the CDC determined that it could rely upon approval of the Institutional Review Board of the Fundacion Dominicana de Infectologia, Inc. (Reliance for approval reference no: 5867).

## 3. Results

### 3.1 Clinical characteristics and aetiology

A total of 121 case-patients were enrolled. The median age was 31 months, with a range from 1 week to 14 years. Among 120 case-patients with available sex, 81 (67.5%) were male. Underlying chronic illness was reported for 16 (13.2%) case-patients, including asthma (*n* = 7), sickle cell anaemia (*n* = 6), trisomy 21 (*n* = 1), anaemia (*n* = 1), and tuberculosis (*n* = 1). Two patients were diagnosed with other major illnesses during their hospitalisation (one with Burkitt’s lymphoma and one with a mediastinal tumor). One case-patient died; all others were discharged or remained hospitalised (*n* = 5) at last follow-up. Among 110 case-patients with data available on prior medication use, 60 (54.5%) had previously received antibiotics for the current illness.

All 121 case-patients had pleural fluid culture performed, and 112 (92.6%) had pleural fluid specimens tested by PCR. An aetiology was determined by culture and/or PCR for 85 (70.2%) cases (Table 1). The leading cause was *S. pneumoniae*, which was detected in 62 (51.2%) cases. The second most common aetiology was *S. aureus* (*n* = 19, 15.7%); 3 (15.8%) isolates were methicillin-resistant. Less common aetiologies detected included *S. pyogenes* (*n* = 2), *S. mitis* (*n* = 1), and *Candida* spp. (*n* = 1). Among the 112 samples tested by PCR, no RSV or human rhinovirus was detected.

**Table 1:** Aetiology of paediatric pneumonia with effusion based on results of pleural fluid testing by culture and polymerase chain reaction

Organism	Detected by culture^a^		Detected by PCR^b^		Detected by culture and/or PCR	
	*n*	(%)	*n*	(%)	*n*	(%)
*Streptococcus pneumoniae* ^c^	19	(15.7)	61	(54.5)	62	(51.2)
*Staphylococcus aureus* ^c^	19	(15.7)	--		19	(16.7)
*Streptococcus pyogenes*	1	(0.8)	2	(1.8)	2	(1.7)
*Streptococcus mitis*	1	(0.8)	--		1	(0.8)
*Candida* spp.	1	(0.8)	--		1	(0.8)
No aetiology determined	81	(66.9)	49	(43.8)	36	(29.8)

PCR, polymerase chain reaction

^a^121 tested by culture

^b^112 tested by PCR

^c^Includes one *S. pneumoniae* / *S. aureus* co-infection

**Figure 1:**
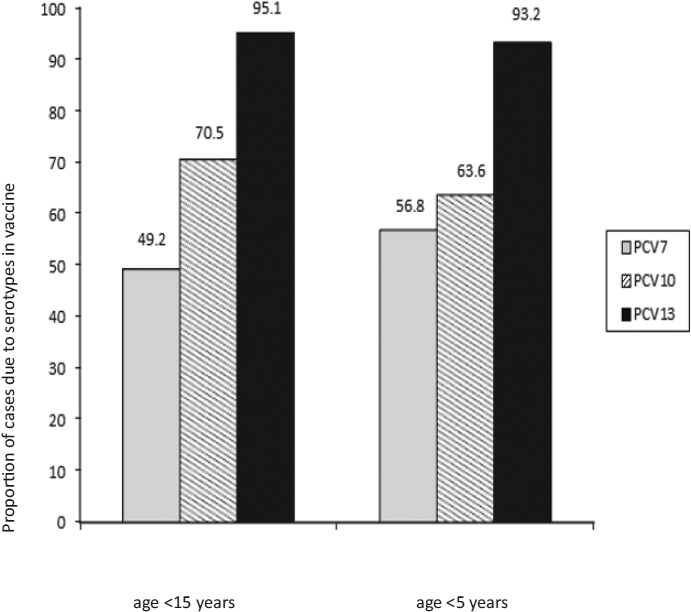
Serotype coverage of the 7-, 10- and 13-valent pneumococcal conjugate vaccines (PCVs) among children with pneumococcal pneumonia with effusion (*n* = 61)

*S. pneumoniae* was cultured from pleural fluid in 19 (30.6%) cases with a pneumococcal aetiology, and 18 of those isolates were tested for antimicrobial susceptibility. One (5.6%) isolate had intermediate susceptibility to both penicillin and ceftriaxone, 3 (16.7%) additional isolates had intermediate susceptibility to ceftriaxone, and the others were fully susceptible to both antibiotics. Among the 62 case-patients with *S. pneumoniae* infections, 61 had specimens available for serotyping by PCR, and 60 (98.4%) were serotyped (Table 2). The most frequent serotype was 14 (*n* = 20, 32.8%), followed by serotypes 1 (*n* = 13, 21.3%), 3 (*n* = 12, 19.7%), and 6A/B (*n* = 6, 9.8%). All were single serotype infections with the exception of one specimen in which serotypes 19A and 5 were detected. Among all children with pneumococcal pneumonia with effusion, the proportion due to serotypes included in the 10- and 13-valent PCV were 70.5% and 95.1%, respectively (Figure 1). Based on this serotype coverage and the proportion of cases with a pneumococcal aetiology, the 10- and 13-valent PCV could reduce the burden of pneumonia with effusion by 36% and 49%, respectively.

**Table 2:** Serotypes detected in children with pneumococcal pneumonia with effusion, *n* = 61^a^

Serotype	*n*	(%)
14	20	(32.8)
1	13	(21.3)
3	12	(19.7)
6A/6B^b^	6	(9.8)
23F	3	(4.9)
19A^c^	3	(4.9)
15B/15C^b^	2	(3.3)
5^c^	1	(1.6)
9V/9A^b^	1	(1.6)
Not typeable for 40 serotypes by PCR	1	(1.6)

PCR, polymerase chain reaction

^a^Among 62 case-patients with *Streptococcus pneumoniae* detected in the pleural fluid, 61 had specimens available for serotyping

^b^Serotyping by PCR does not distinguish between the following serotypes within the same serogroup: 6A and 6B, 15B and 15C, 9V and 9A

^c^Includes one co-infection with serotypes 19A and 5

### 3.2 Detection of S. pneumoniae by ICT and culture

**Figure 2:**
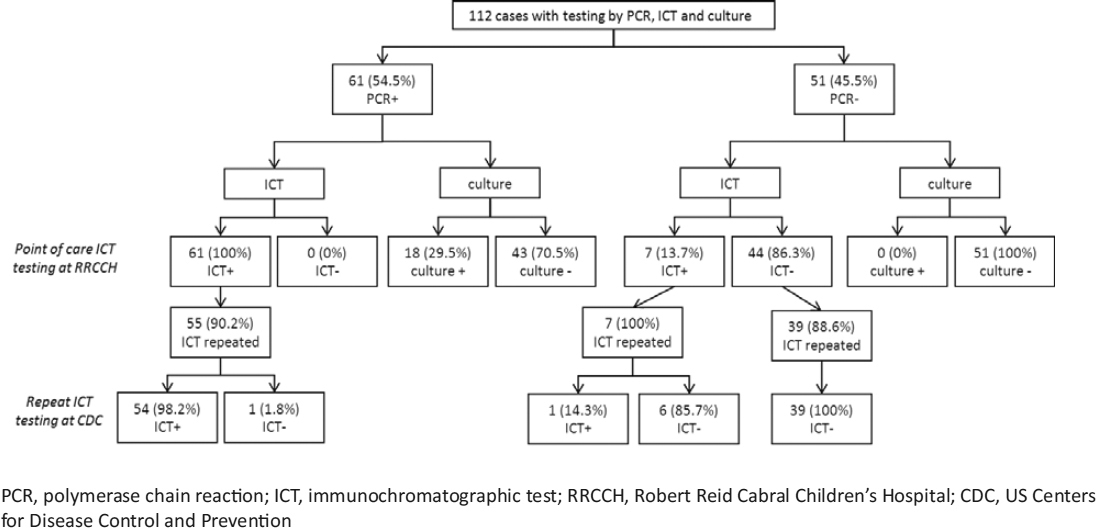
Detection of *Streptococcus pneumoniae* by polymerase chain reaction, immunochromatographic test and culture

Pleural fluid samples from 112 case-patients were tested for *S. pneumoniae* by PCR, ICT, and culture (Figure 2). PCR for *S. pneumoniae* was positive in 61 samples; all were also positive by point of care ICT at RRCCH. Of the 51 samples that were PCR-negative for *S. pneumoniae*, 7 (13.7%) were positive by ICT at RRCCH. ICT was repeated at CDC for 101 (90.2%) samples. Compared with PCR results, ICT testing at CDC yielded 1 false-negative and 1 false-positive result. The PCR-negative sample that was positive by ICT at CDC was also positive by ICT at RCCH. The other 6 samples that had false-positive ICT results at RCCH were negative when re-tested by ICT at CDC; one grew *S. aureus* from culture, the others had no organisms detected. Culture detected *S. pneumoniae* in 18 (29.5%) of the 61 cases that were positive by PCR.

The sensitivity of point of care ICT compared with PCR (Table 3) was 100% (95% confidence interval [CI] 94.1%–100%), while the specificity was 86.3% (95% CI 73.7%–94.3%). The sensitivity, specificity, positive and negative predictive values of ICT performed in the CDC laboratory were all greater than 95%. The performance of ICT was not affected by prior antibiotic use. The sensitivity of culture (29.5%, 95% CI 18.5%–42.6%) was significantly lower than that of ICT, and further declined in the context of prior antibiotic use (9.4%, 95%CI 2.0%–25.0%).

**Table 3:** Performance of immunochromatographic test and culture compared with polymerase chain reaction to detect *Streptococcus pneumoniae* in pleural fluid

Test	Sensitivity (95% CI)	Specificity (95% CI)	Positive predictive value (95% CI)	Negative predictive value (95% CI)
Point of care ICT at RRCCH				
All samples (*n* = 112)	100 (94.1–100)	86.3 (73.7–94.3)	89.7 (80.0–95.8)	100 (92.0–100)
Samples from children with prior antibiotic use^a^ (*n* = 58)	100 (89.1–100)	92.3 (74.9–99.0)	94.1 (80.3–99.3)	100 (85.8–100)
Repeat ICT at CDC				
All samples^b^ (*n* = 101)	98.2 (90.3–100)	97.8 (88.5–99.9)	98.2 (90.3–100)	97.8 (88.5–99.9)
Samples from children with prior antibiotic use^a^ (*n* = 53)	96.6 (82.2–99.9)	100 (85.8–100)	100 (87.8–100)	97.8 (88.5–99.9)
Culture				
All samples (n=112)	29.5 (18.5–42.6)	100 (93.0–100)	100 (81.5–100)	54.3 (43.7–64.6)
Samples from children with prior antibiotic use^a^ (*n* = 58)	9.4 (2.0–25.0)	100 (86.8–100)	100 (29.2–100)	47.3 (33.6–61.2)

ICT, immunochromatographic test; RRCCH, Robert Reid Cabral Children’s Hospital; CI, confidence interval; CDC, US Centers for Disease Control and Prevention

^a^Analysis was restricted to children within each group who reported prior antibiotic use

^b^101 of the 112 samples were available for repeat ICT at the CDC

## 4. Discussion

This study has demonstrated an important burden of pneumococcal disease among children with pneumonia with effusion in the Dominican Republic. *S. pneumoniae* was detected in more than half of all cases. This finding is consistent with other studies that have reported *S. pneumoniae* to be the leading cause of pneumonia with effusion [16–19]. *S. aureus* was the second most common aetiology identified in this study. Because *S. aureus* was detected by culture only (not by PCR), the true contribution of this pathogen was likely greater than what was observed. Some studies of paediatric pneumonia with effusion and empyema have found *S. aureus* to be the leading cause [20–22], and *S. pyogenes*, which was found in 2 cases in this study, has also been found to be an important aetiology [16, 17, 22]. We did not identify any cases due to RSV, which is a leading cause of severe respiratory illness among young children [23], or human rhinovirus, which is among the most commonly detected respiratory pathogens in this age group [24]. Interestingly, a recent paper from Brazil described probable viral aetiologies among 9 of 18 children with pneumonia with effusion [25]; however the viruses were detected in the nasopharynx or based on serologic tests, and pleural fluid samples (which were available for a minority of case-patients) were tested by culture only, thus the results are not directly comparable to our findings. Other studies have found only bacterial infections to be significantly associated with pleural effusion [26, 27]. Our findings similarly do not support an important role of viruses in paediatric pneumonia with effusion, and highlight the primary role of *S. pneumoniae*.

These data suggest that the 13-valent PCV, which was introduced in the national immunisation program in the Dominican Republic in August 2013, could reduce the burden of pneumonia with effusion in children by up to 49%. The 7-valent PCV, which has been available since 2000, is highly effective against disease caused by vaccine serotypes [28] and led to a 39% decline in pneumonia hospitalisation among children <2 years in the United States [29]. Rates of empyema in children, however, have increased in the US and other locations since the 1990s [30, 31] — a trend which has not abated despite the introduction of the 7-valent PCV [30, 32]. Serotype 1, which appears to have been a contributing factor to the rise in pneumonia with effusion in some settings [4], is not included in the 7-valent vaccine but is included in both the 10- and 13-valent formulations currently available. The results of studies evaluating immunogenic responses to the 10- and 13-valent PCVs suggest that they will protect against serotype 1 [33, 34], but data on effectiveness against clinical outcomes are not yet available. Serotype 1 was the second most common pneumococcal serotype identified in this study. It will be important to continue to monitor trends in pneumonia with effusion and pneumococcal serotype distribution in the Dominican Republic in the post-PCV13 era.

Our findings contribute to a growing body of evidence on the utility of ICT for detecting *S. pneumoniae* in pleural fluid. The rapid, easy-to-use assay was originally developed for detection of pneumococcal antigen in urine samples from adults [35]; however, studies have examined its performance when testing other specimens such as middle ear fluid [36, 37], cerebrospinal fluid [38] and pleural fluid [16, 39–43]. The high sensitivity and specificity we found for ICT performed on pleural fluid specimens are consistent with those reported in the published literature — which both range from 71% to 100% [16, 19, 39–44]. Similar to those studies, we found the sensitivity of ICT to be much greater than that of culture, particularly for samples from patients on antibiotics. We found 6 samples that were positive by ICT when used as a point of care test and negative when the ICT was repeated at CDC. These discrepancies may represent false-positive results on the initial test; false-positive results on ICT have been reported in the presence of other streptococcal species, anaerobes and *Enterococcus faecalis* [43, 44]. In our study, one case with a false-positive ICT result had a pleural fluid culture that grew *S. aureus*. Alternatively the discrepancies could be due to errors in point of care testing. Yet overall, there were very few discrepant results, confirming the useful role that ICT can play in measuring the burden of pneumococcal pneumonia.

This study had several limitations. RRCCH is a referral hospital, so children admitted there may represent the more severe or complex end of the spectrum of disease. There is no defined population denominator, so it is not possible to calculate incidence to estimate the population-level burden of paediatric pneumonia with effusion. And while we tested for the most common pathogens as well as some less common causes, there are other causes of pleural effusion that were not tested for (e.g. tuberculosis, other viruses) or that were tested for by culture rather than more sensitive molecular assays.

Nonetheless, this study provides important insight on the burden of pneumonia with effusion in a middle-income Latin American country. It highlights the importance of *S. pneumoniae*, and provides an estimate of the potential impact of PCV introduction on pneumococcal, and overall, pneumonia with effusion in children. We also demonstrated the utility of ICT for detecting pneumococcal disease in children with pneumonia and pleural effusion. The ICT can play an important role both clinically, as a point of care test, and epidemiologically, as a tool for measuring the burden of pneumococcal disease and evaluating the impact of PCV introduction. Such data can inform policy decisions regarding the introduction and sustained use of PCV, which will ultimately help reduce illness and death due to pneumonia among children in the Dominican Republic and elsewhere.
